# Demographic and Psychosocial Characteristics Associated With Use of a Prostate Cancer Survivorship Website: Implications From a Multisite Randomized Controlled Trial

**DOI:** 10.2196/27890

**Published:** 2022-03-21

**Authors:** Allison Marziliano, Michael A Diefenbach, Shawna V Hudson, Erin K Tagai, Elizabeth A Handorf, Alicja Bator, Suzanne M Miller

**Affiliations:** 1 Center for Health Innovations and Outcomes Research Feinstein Institutes for Medical Research Northwell Health Manhasset, NY United States; 2 Rutgers Robert Wood Johnson Medical School Rutgers, The State University of New Jersey New Brunswick, NJ United States; 3 Cancer Prevention and Control Fox Chase Cancer Center/Temple University Health System Philadelphia, PA United States

**Keywords:** prostate cancer, cancer survivorship, web-based resource, monitoring style of coping, cancer, survivorship, eHealth, emotions, interpersonal

## Abstract

**Background:**

Many prostate cancer (PC) survivors experience disease and treatment-related symptomatology in both the physical and psychosocial domains. Although the benefits and barriers to using web-based resources for cancer patients are well-documented, less research has focused on the personal characteristics important for efficient tailoring and targeting of information that are associated with usage.

**Objective:**

We used the Cognitive-Social Health Information Processing (C-SHIP) framework to guide our exploration of personal characteristics associated with use of PROGRESS, an informational PC survivorship website that addresses physical, emotional, interpersonal, and practical concerns relevant for PC survivors.

**Methods:**

PC survivors (N=217) were randomized to the intervention arm (PROGRESS) of a randomized controlled trial. Of those randomized to the intervention arm, 84 used PROGRESS, and 133 did not use PROGRESS. Multivariable analyses evaluated demographic and psychosocial characteristics (eg, style of coping, health literacy, self-efficacy, affective states of depression, anxiety, and fatigue) associated with website use.

**Results:**

A larger proportion of non-Hispanic White (68/160, 42.5%), compared with non-Hispanic Black (9/40, 23%), participants used PROGRESS (*P*<.001). Further, PROGRESS users were older in age (*P*<.001), had a monitoring style of coping (*P*=.01), and were less depressed (*P*=.004), anxious (*P*=.02), and fatigued (*P*<.001) than nonusers. Education, income, health literacy, blunting style of coping, self-efficacy, and treatment type (radiation therapy or surgery) were not significantly related to use. On multivariable analyses, race (OR 0.28, *P*<.001), age (OR 1.05, *P*<.001), monitoring style of coping (OR 1.27, *P*=.02), and overall mood (OR 0.98, *P*<.001) remained significant.

**Conclusions:**

A combination of monitoring and low levels of negative affect were associated with website use. Additionally, users were older, non-Hispanic White survivors. To ensure that important survivorship-relevant information reaches users, future efforts need to focus on enhancing patient engagement.

**Trial Registration:**

ClinicalTrials.gov NCT02224482; https://clinicaltrials.gov/ct2/show/NCT02224482

## Introduction

Prostate cancer (PC) is the second most common cancer diagnosed in men in the United States, with about 1 in 9 men diagnosed during their lifetime. The American Cancer Society estimates that, in 2021, there will be 248,530 new cases of PC in the United States [1]. The 5-year relative survival rate for localized PC is near 100% [1]. Consequently, the population of PC survivors is growing, exhibiting specific disease and treatment-related symptomatology in both the physical (eg, urinary, bowel, or sexual dysfunction [2,3]) and psychosocial (eg, high rates of distress, anxiety, reduced quality of life, depression, adjustment difficulties, fear of cancer recurrence [4-7]) domains. Further, studies have shown there is a significant interpersonal impact of PC on patients’ relationships with their spouses and loved ones, as they struggle with intimacy, sexual confidence, sense of masculinity, familial cancer risk, and communicating about their diagnosis, treatment, and symptoms with friends [8,9].

The rapid development of modern technology has facilitated the use of web-based resources for individuals dealing with illness and treatment-related side effects, as is the case for PC survivors. Web-based resources have been developed and evaluated for many groups including breast cancer survivors [10,11], patients with melanoma [12], and families with parental cancer, as well as to educate nurses on reproductive issues in cancer patients [13]. Existing web-based resources specifically for patients with or survivors of PC include education or decision aids, interventions to reduce distress after treatment, and physical activity interventions [13-21].

Benefits to using web-based resources include ease of access at the patient’s own schedule in a private place; ability to access the intervention through multiple channels (ie, personal computer, tablet, smartphone); augmented content through interactive videos, graphics, and testimonials; tailored content for treatment approaches or specific time points in the recovery trajectory; access transcending geographical barriers; and tracking patient recovery in real time [22-28]. Yet, there are also unique barriers, including lack of internet access, participants’ privacy concerns, and program costs associated with developing and maintaining web-based resources [29-32]. In addition, studies have reported challenges engaging patients for initial access and staying engaged in recommended programs. A systematic review of adherence to web-based interventions showed that, on average, only about 50% of participants engage in software-based interventions, with variations from 10% to 90% across studies [33].

To increase engagement and persistent use of health-related software programs, it is therefore important to identify psychosocial characteristics that go beyond the commonly known access and demographic variables (eg, younger age, higher education) [28,29]. Thus, the current study aimed to identify patient demographic and psychosocial characteristics associated with use of PROGRESS, a web-based intervention for PC survivors.

## Methods

### Conceptual Framework

Our study is guided by the Cognitive-Social Health Information Processing (C-SHIP) model, a theoretical framework that identifies 5 cognitive-affective constructs that are associated with engagement in health protective behaviors [34,35]. These constructs consist of (1) cancer-relevant interpretations, (2) beliefs and expectations about cancer treatment and disease outcomes, (3) cancer-relevant goals and values, (4) cancer-relevant affective states, and (5) self-regulatory competencies and skills for generating and maintaining goal-oriented health-related behaviors. For the purpose of our analyses, these theoretical C-SHIP constructs were operationalized with the following patient-level variables: monitoring styles of coping (ie, the disposition to stand for and attend to health-relevant cues that entail C-SHIP’s cancer-relevant interpretations, beliefs, and expectations about health risks); health literacy (C-SHIP’s skills for generating and maintaining goal-oriented health behaviors); self-efficacy (C-SHIP’s self-regulatory competencies); depression, anxiety, and fatigue (C-SHIP’s cancer-relevant affective states). We hypothesized that these constructs are significantly and positively related to patient engagement and usage of the PROGRESS program.

### Patient Recruitment

For the parent randomized controlled trial (RCT), PC patients were recruited during routine posttreatment follow-up appointments at 4 mid-Atlantic cancer centers. Recruitment occurred over the course of 3 years (2013-2016). Patients were eligible if they were diagnosed with localized PC (T1-T3c N0M0), were within 1 year of treatment completion, had regular access to a computer or a tablet with internet either at home or at another public place, were aged 18 years or older, were able to give consent, and were able to communicate in English. Exclusion criteria were presence of another primary cancer or a cancer recurrence.

Eligible patients who agreed to participate in the study were enrolled after signing the consent form and completing the baseline survey. Using block randomization by site, participants were randomized to either the control group (print materials: NCI’s *Facing Forward: Life after Cancer Treatment* and *What You Need to Know about Prostate Cancer*) or the intervention group (PROGRESS + print materials). This manuscript focuses only on the subgroup of participants that were randomized to the intervention group, PROGRESS. This study was approved by the Institutional Review Boards at Fox Chase Cancer Center (#11‐825), Rutgers University (#0220110092), Northwell Health (#14‐672), and Mt. Sinai (#11‐01136).

### Intervention Condition

#### PROGRESS Content

PROGRESS is a self-paced, web-based educational program to address PC survivors’ information needs in 6 specific domains suggested by prior work of the investigators [15,16,21,36-37], literature on this population, and formative work to develop the intervention content. These domains are (1) treatment type and expected prostate specific antigen (PSA) changes, which indicate PC status and progression; (2) physical side effects (eg, urinary and sexual dysfunction); (3) emotional concerns (eg, fear of cancer recurrence); (4) interpersonal concerns (eg, communications with providers and family); (5) practical concerns (eg, follow-up care, financial needs); and (6) healthy lifestyle (eg, nutrition, physical activities) [15,38]. Information is culturally targeted and tailored to different survivorship stages (eg, short-term vs long-term survivorship needs). PROGRESS was developed through a 2-phase, qualitative formative research study. Phase 1 included individual interviews with 5 and group interviews with 12 early-stage prostate cancer patients to determine intervention content and interface. Phase 1 employed iterative user and usability testing (n=12) to finalize the intervention. Participants expressed interest in action-oriented content on managing treatment side effects, handling body image and comorbidities related to overweight or obesity, coping with emotional and communication issues, tips to reduce disruption of daily living activities, and health skills training tools. An extensive readability evaluation was conducted in order to ensure that the PROGRESS intervention met plain language standards. To conduct this evaluation, members of the Office of Health Communications and Health Disparities at Fox Chase Cancer Center used the software program, Health Literacy Advisor, to calculate reading grade levels and offer replacement text for complex terms and long sentences. All text was revised as needed to conform to an 8th or 9th grade target level reading range. For more information on the development, preliminary testing, and efficacy of PROGRESS, see Miller et al [38] and Tagai et al [39,40].

PROGRESS features include a topics tab (addressing financial or legal issues; interpersonal communication, emotional and practical concerns; negative feelings; and side effects); videos from physicians, patients, and content experts; fields for personal tracking (of PSA level, health status, weight, sleep, urinary or erectile dysfunction, medication, living habits, and questions for upcoming physician appointments); information on the latest PC findings (in prevention, screening, treatment, and survivorship); a virtual health center and navigator; theoretically guided normalizing messages; testimonials from a group of diverse PC survivors; and technology support (a tutorial program and a help desk). The software program underwent extensive usability testing before it was released for study purposes [38].

#### Translating Theory-Driven Constructs to Practical Content

The 5 key theoretical constructs were operationalized within PROGRESS through the program’s components of (1) providing accurate information, (2) creating realistic expectations and promoting self-efficacy, (3) exploring the patient’s goals and values and encouraging behavior consistent with them, (4) validating feelings and facilitating emotional support, and (5) providing information and training to maximize self-regulatory competencies and skills.

### Data Collection

Assessments were conducted at baseline and at 1-month, 3-month, and 6-month follow-ups. Data for these analyses were drawn from the baseline assessment and indication of website use during the study. Patients completed the assessments via paper-and-pencil survey, online via REDCap, or telephone interview. Follow-up telephone calls or emails (based on participant’s preferred survey completion method) were used for noncompleted surveys. Participants received a US $20 gift card for each completed assessment.

### Study Measures

Demographics assessed at baseline included race/ethnicity, age, and education. Comorbidities were captured using the Charlson Comorbidity Index, a 16-item weighted measure evaluating the presence or absence, or severity, of illnesses [41]. The Charlson is scored as a weighted sum of the illnesses, such that a positive response for certain illnesses (ie, myocardial infarction, congestive health failure, dementia) adds 1 point each, a positive response for other illnesses (ie, leukemia) adds 2 points each, and a positive response for AIDS adds 6 points. The remaining illnesses, which are scored on a severity scale, are multiplied by 2, 3, 2, and 6 for diabetes mellitus, liver disease, renal disease, and malignant solid tumor, respectively. Style of coping was assessed using the Monitor/Blunter Style Scale (MBSS) [42]. Health Literacy was assessed using a 3-item screen for health literacy [43,44]. The 3 items are: “How often do you have someone help you read hospital materials?”, “How confident are you filling out medical forms by yourself?”, and “How often do you have problems learning about your medical condition because of difficulty understanding written information?” Response options are on a 5-point Likert scale ranging from “always to never” or “extremely to not at all.” Self-efficacy for symptom control was measured using an author-constructed 12-item scale that asked participants how confident they were to “manage any treatment-related fatigue,” “recover your emotional well-being,” and “does your family know how to support you?” [45]. Depression, anxiety, and fatigue were assessed with the Profile of Mood States Short Form (POMS-SF), using the respective depression, anxiety, and fatigue subscales [46]. Depression was also assessed with the Center for Epidemiological Studies-Depression subscale (CES-D) [47]. Undergoing surgery or radiation therapy was assessed via self-report and confirmed with medical chart abstraction.

The outcome variable, PROGRESS use, was a binary variable coded as “use” or “nonuse” and was obtained via Google Analytics after completion of the participants’ last follow-up assessments. Participants randomized to PROGRESS were considered to have used PROGRESS if they clicked beyond the home page at least once during the study period. Participants were categorized as nonusers if they did not log in or logged in but did not click through to any other page beyond the home page. We were unable to track amount of website use with the available tracking metrics.

### Statistical Analyses

Data were analyzed with SPSS version 19.0 and R version 3.6. Descriptive statistics were used to characterize the sample [48,49]. We then used a series of univariable logistic regression models to test the association between website use and each variable of interest, using generalized estimating equations with robust standard errors to account for within-site correlation. We also used a multivariable logistic regression model to simultaneously evaluate the variables’ associations with website use. Significant variables from the univariate logistic regression analyses, a priori variables of interest, and surgery or radiation treatment (hormone therapy was excluded due to low number of patients receiving this treatment) were included in the multivariable logistic regression model. To avoid collinearity, the POMS-SF total score was included rather than the individual subscales. A 2-sided *P* value <.05 was considered statistically significant.

## Results

### Recruitment

**Figure 1:**
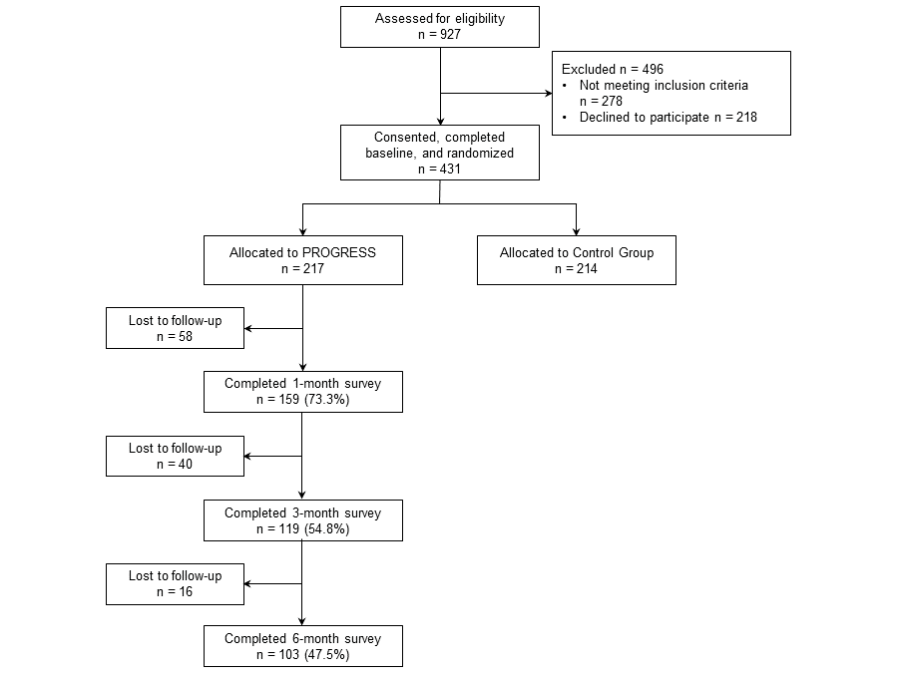
CONSORT (Consolidated Standards of Reporting Trials) diagram for the PROGRESS intervention arm.

A total of 927 participants were assessed for eligibility; 278 did not meet inclusion criteria, leaving 649 eligible. A total of 431 participants (66.4% of those eligible) consented and were enrolled and randomized (217 PC survivors were randomized to PROGRESS, and 214 were randomized to the control condition), and 218 declined to participate. Of those randomized to PROGRESS, 73.3% (159/217) completed the 1-month time point, 54.8% (119/217) completed the 3-month time point, and 47.5% (103/217) completed the 6-month time point. See Figure 1 for a detailed CONSORT (Consolidated Standards of Reporting Trials) diagram, adapted for this study focusing on the PROGRESS intervention arm.

### Sample Demographics

Overall, the sample was mostly non-Hispanic White (160/217, 73.7%), married (175/217, 80.6%), and with no comorbidities (167/214, 78.0%). About half (103/215, 47.9%) had a college degree or higher, and about half (115/217, 53.0%) endorsed that they were in the highest income bracket (US $75,001 and greater). In terms of treatment, about half (112/217, 51.6%) had surgery. The average age of the sample was 63.79 (SD 6.67) years. See Table 1 for details.

Participants’ mean scores were 2.46 (SD 2.20) and 1.44 (SD 1.41) on the monitoring and blunting subscales, respectively; 13.12 (SD 2.24) on the measure of health literacy; 8.75 (SD 1.11) on the self-efficacy for re-entry scale; 4.76 (SD 4.32) on the anxiety measure; and 6.27 (SD 5.05) on the measure of fatigue. Further, the average scores on the POMS-SF depression measure and CES-D measure were 3.30 (SD 3.94) and 5.99 (SD 5.27), respectively. See Table 1 for details.

**Table 1:** Demographic characteristics of website users and nonusers (controlling for study site).

Variable			Website users (n=84)		Nonusers (n=133)		Total sample (N=217)		*P* value
**Race/ethnicity, n (%)**									
	Non-Hispanic White	68 (81.0)		92 (69.2)		160 (73.7)		ref^a^	
	Non-Hispanic Black	9 (10.7)		31 (23.3)		40 (18.4)		<.001	
	All other races	7 (8.3)		10 (7.5)		17 (7.8)		.86	
Age (years), mean (SD)			65.37 (7.03)		62.79 (6.27)		63.79 (6.67)		<.001
**Education^b^, n (%)**									
	High school or less	16 (19.0)		33 (25.2)		49 (22.8)		ref	
	Some college	26 (31.0)		37 (28.2)		63 (29.3)		.40	
	College degree	22 (26.2)		33 (25.2)		55 (25.6)		.08	
	Graduate/professional degree	20 (23.8)		28 (21.4)		48 (22.3)		.60	
**Household income (US $), n (%)**									
	<45,000	11 (13.1)		25 (18.8)		36 (16.6)		ref	
	45,001-75,000	21 (25.0)		31 (23.3)		52 (24.0)		.34	
	≥75,001	49 (58.3)		66 (49.6)		115 (53.0)		.25	
	Missing	3 (3.6)		11 (8.3)		14 (6.5)		.30	
**Marital status, n (%)**									
	Never married, divorced, separated, widowed, single, unknown, refused	12 (14.3)		30 (22.6)		42 (19.4)		ref	
	Married or domestic partnership	72 (85.7)		103 (77.4)		175 (80.6)		.08	
Health literacy, mean (SD)			13.51 (1.94)		12.87 (2.39)		13.12 (2.24)		.18
Blunting, mean (SD)			1.65 (1.24)		1.31 (1.50)		1.44 (1.41)		.29
Monitoring, mean (SD)			3.01 (1.93)		2.11 (2.29)		2.46 (2.20)		.01
Self-efficacy for re-entry, mean (SD)			8.83 (0.98)		8.70 (1.18)		8.75 (1.11)		.07
Depression (POMS-SF^c^), mean (SD)			2.89 (3.25)		3.56 (4.31)		3.30 (3.94)		.15
Depression (CES-D^d^), mean (SD)			5.29 (4.17)		6.43 (5.84)		5.99 (5.27)		<.001
Tense/anxiety, mean (SD)			4.36 (3.74)		5.01 (4.65)		4.76 (4.32)		.02
Fatigue, mean (SD)			5.72 (4.64)		6.61 (5.28)		6.27 (5.05)		<.001
**Charlson Comorbidity Index^e^, n (%)**									
	0	66 (79.5)		101 (77.1)		167 (78.0)		ref	
	1	10 (12.1)		18 (13.7)		28 (13.1)		.52	
	≥2	7 (8.4)		12 (9.2)		19 (8.9)		.30	
**Radiation therapy, n (%)**									
	No	52 (61.9)		96 (72.2)		148 (68.2)		.09	
	Yes	32 (38.1)		37 (27.8)		69 (31.8)			
**Surgery, n (%)**									
	No	39 (46.4)		66 (49.6)		105 (48.4)		.80	
	Yes	45 (53.6)		67 (50.4)		112 (51.6)			

^a^ref: reference.

^b^Total n=215.

^c^POMS-SF: Profile of Mood States Short Form.

^d^CES-D: Center for Epidemiological Studies-Depression subscale.

^e^Total n=214.

### Main Analyses

Of the 217 patients, 84 (38.7%) reported using the website versus the 133 (61.3%) who reported that they did not use the website. When controlling for study site, there were significant differences between those who used PROGRESS and those who did not use PROGRESS in the following variables: race/ethnicity, age, style of coping, depression, anxiety, and fatigue. Specifically, a larger proportion of non-Hispanic White (68/160, 42.5%), compared with non-Hispanic Black (9/40, 23%), participants used PROGRESS (*P*<.001; Table 1). Users of PROGRESS were older in age (mean 65.37, SD 7.03 years vs mean 62.79, SD 6.27 years; *P*<.001), higher on monitoring (mean 3.01, SD 1.93 vs mean 2.11, SD 2.29; *P*=.01), and less depressed (mean 5.29, SD 4.17 vs mean 6.43, SD 5.84; *P*<.001), anxious (mean 4.36, SD 3.74 vs mean 5.01, SD 4.65; *P*=.02), and fatigued (mean 5.72, SD 4.64 vs mean 6.61, SD 5.28; *P*<.001). There were no other significant differences between those who used PROGRESS and those who did not use PROGRESS.

In the multivariable model, non-Hispanic Black participants were significantly less likely to use the website than non-Hispanic White participants (OR 0.28, 95% CI 0.25-0.32; Table 2). There was no significant difference between non-Hispanic White participants and those of all other race/ethnicities (OR 0.77, 95% CI 0.43-1.36). Additionally, older participants were more likely to use the website (OR 1.05, 95% CI 1.04-1.07), as well as those reporting greater monitoring style of coping (OR 1.27, 95% CI 1.04-1.56). Finally, those with a more negative mood state were significantly less likely to use the website (OR 0.98, 95% CI 0.97-0.98).

**Table 2:** Multivariable logistic regression analysis (controlling for study site).

Variable	OR^a^	SE	LCL^b^	UCL^c^
Non-Hispanic Black^d^	0.28	.07	0.25	0.32
All other races/ethnicities^d^	0.77	.29	0.43	1.36
Age	1.05	.01	1.04	1.07
Monitoring	1.27	.10	1.04	1.56
Self-efficacy for re-entry	0.89	.07	0.78	1.01
Mood total	0.98	.004	0.97	0.98
Radiation therapy^e^	1.42	.19	0.98	2.07
Surgery^e^	1.19	.39	0.56	2.57

^a^OR: odds ratio.

^b^LCL: lower confidence limit.

^c^UCL: upper confidence limit.

^d^Reference group: non-Hispanic White.

^e^Reference group: did not receive treatment.

## Discussion

### Principal Findings

PROGRESS was more likely to be used by PC survivors who were high on monitoring style of coping, a coping style to deal with threat that involves scanning for and magnifying disease-related cues [42]. Monitors are often more concerned about their illness, experience more treatment-related side effects, are more knowledgeable about their medical situation, feel themselves to be at greater personal risk, and are less satisfied with and more demanding about the psychosocial aspects of their care [50]. Notably, they are more often adherent to medical recommendations and place greater value on health-related information [50]. The PROGRESS website offered patients characterized as monitors authoritative information about PC from providers and testimonials by a diverse group of survivors who are addressing psychosocial aspects of the disease. Thus, PROGRESS offers monitors health-related information that they typically value and that translated into higher use.

Results also showed that PROGRESS was more likely to be used by non-Hispanic, White PC survivors, confirming prior established patterns of internet use. A study conducted by the Pew Research Center indicated that internet usage is more common in White compared with non-Hispanic Black populations [51,52]. Racial disparities in education are well-documented and favor non-minority populations [53,54]. Patients with higher levels of education are more likely to have used a computer in the past and would be more apt to use offered resources, such as PROGRESS.

PROGRESS users’ relatively positive mood (less depressed, less anxious, and less fatigued) allows them to mobilize in support of their health, which includes taking advantage of the information PROGRESS has to offer. Using PROGRESS and other related health resources may be blunted in patients who are highly depressed, as depressed patients often cannot empower themselves to take care of their health. Similarly, high anxiety may hinder the use of PROGRESS, as these patients may have anxiety about the information that PROGRESS will provide. Patients with high levels of fatigue may not feel energized to a degree necessary to use a web-based resource such as PROGRESS. Our results also showed that older patients were more likely to use PROGRESS. Though this finding was statistically significant, it was not clinically significant, with an average age of 65.37 years compared with 62.79 years. It is encouraging that older men are using this web-based resource, as PC survivors more generally are an older demographic given the average age of diagnosis is 66 years old [1]. Analyzing age as a categorical variable produces the same results. It is possible that the older participants were more motivated to use the site or that this finding is due to chance, as the numbers for each individual group are not that large.

The study findings highlight the need for strategies to increase patient engagement with web-based tools, as less than 40% of the intervention group reported that they used PROGRESS. Engagement needs to go beyond the commonly accepted development and access strategies, such as ensuring access to a computer and the internet (eg, providing tablets in clinic during down time), ensuring comfort with using a computer and the internet, using language and terminology appropriate for a low health-literate population, incorporating culturally targeted material into the program, and prompting use through text message or email reminders. The value of the recommended services needs to be clearly communicated, and if possible, gamification elements, such as badges or virtual competition with other users, can be incorporated.

Indeed, our study team did engage in a thoughtful, iterative process to design the PROGRESS website that included stakeholder feedback. Based on the initial design phase and the usability testing, the PROGRESS site was very positively evaluated. We think that this highlights the need for ongoing usability and acceptability testing, rather than collecting these data at one time point prior to intervention launch. This experience has suggested that, for future studies, researchers should build in a regular review of usage, usability, and acceptability and devise a plan for how to handle the responses if certain intervention components are not well-received. Collecting these data longitudinally during the RCT phase may not be able to inform the intervention being tested, but it would be useful to inform future interventions.

Low patient engagement is a threat to efforts to evaluate the efficacy of web-based tools. Before beginning a research study, power calculations are needed to ascertain sample size requirements necessary to detect clinically meaningful differences. Understanding that all of those enrolled in the intervention arm may not actually engage with the intervention may alter researchers’ plans for how many patients to enroll. On a related note, researchers may, a priori, plan to conduct their analyses in 2 ways: first, comparing intervention and control, and second, comparing within the intervention arm, users with nonusers. In addition, rather than “using website” as a simple binary variable, future research should employ more sophisticated website use tracking features that allow investigators to capture detailed website use, such as time spent with each page, pages with most views, and number of downloads.

### Limitations

This study is not without its limitations. First, the sample was highly homogenous and was mostly non-Hispanic White and high income, and thus, the results may not be generalizable to other, more diverse patient groups. Second, our “website use” variable was binary and therefore did not allow us to evaluate the full continuum of website usage from those who never logged in to the super-users who used PROGRESS over and over again. These limitations aside, we believe our study substantially contributes to the literature characterizing the patient profile of a web-based resource user.

### Conclusions

Our study showed that, compared with nonusers, users of PROGRESS, a website for PC survivors, were more likely to be non-Hispanic White (compared with non-Hispanic Black participants), be older in age, have a higher monitoring style of coping, and have experienced higher levels of positive mood. Improved engagement features need to be developed and evaluated to increase the perceived value to patients. Additionally, the existence of a user patient profile indicates the potential to tailor web-based resources accordingly.

## Abbreviations

CES-D: Center for Epidemiological Studies-Depression subscale
CONSORT: Consolidated Standards of Reporting Trials
C-SHIP: Cognitive-Social Health Information Processing
MBSS: Monitor/Blunter Style Scale
PC: prostate cancer
POMS-SF: Profile of Mood States Short Form
PROGRESS: Prostate Cancer Survivorship Website
PSA: prostate specific antigen
RCT: randomized controlled trial

## References

[1] Key Statistics in Prostate Cancer. American Cancer Society.

[2] Ávila M, Patel L, López S, Cortés-Sanabria L, Garin O, Pont À, Ferrer F, Boladeras A, Zamora V, Fosså S, Storås AH, Sanda M, Serra-Sutton V, Ferrer M (2018). Patient-reported outcomes after treatment for clinically localized prostate cancer: A systematic review and meta-analysis. Cancer Treat Rev.

[3] Chen C, Chen Z, Wang K, Hu L, Xu R, He X (2017). Comparisons of health-related quality of life among surgery and radiotherapy for localized prostate cancer: a systematic review and meta-analysis. Oncotarget.

[4] De Sousa A, Sonavane S, Mehta J (2012). Psychological aspects of prostate cancer: a clinical review. Prostate Cancer Prostatic Dis.

[5] Sharpley CF, Bitsika V, Christie DRH (2018). "The Worst Thing Was…": prostate cancer patients' evaluations of their diagnosis and treatment experiences. Am J Mens Health.

[6] Deimling GT, Bowman KF, Sterns S, Wagner LJ, Kahana B (2006). Cancer-related health worries and psychological distress among older adult, long-term cancer survivors. Psychooncology.

[7] Bellizzi KM, Latini DM, Cowan JE, DuChane J, Carroll PR (2008). Fear of recurrence, symptom burden, and health-related quality of life in men with prostate cancer. Urology.

[8] Campbell LC, Keefe FJ, Scipio C, McKee DC, Edwards CL, Herman SH, Johnson LE, Colvin OM, McBride CM, Donatucci C (2007). Facilitating research participation and improving quality of life for African American prostate cancer survivors and their intimate partners. A pilot study of telephone-based coping skills training. Cancer.

[9] Harden J, Northouse L, Cimprich B, Pohl JM, Liang J, Kershaw T (2008). The influence of developmental life stage on quality of life in survivors of prostate cancer and their partners. J Cancer Surviv.

[10] Irene Su H, Stark S, Kwan B, Boles S, Chingos D, Ehren J, Gorman JR, Krychman M, Romero SAD, Mao JJ, Pierce JP, Natarajan L (2019). Efficacy of a web-based women's health survivorship care plan for young breast cancer survivors: a randomized controlled trial. Breast Cancer Res Treat.

[11] Ridner SH, Dietrich MS, Davis AJ, Sinclair V (2020). A randomized clinical trial comparing the impact of a web-based multimedia intervention versus an educational pamphlet on patient outcomes in breast cancer survivors with chronic secondary lymphedema. J Womens Health (Larchmt).

[12] Coups EJ, Manne SL, Ohman Strickland P, Hilgart M, Goydos JS, Heckman CJ, Chamorro P, Rao BK, Davis M, Smith FO, Thorndike FP, Ritterband LM (2019). Randomized controlled trial of the mySmartSkin web-based intervention to promote skin self-examination and sun protection behaviors among individuals diagnosed with melanoma: study design and baseline characteristics. Contemp Clin Trials.

[13] Quinn GP, Bowman Curci M, Reich RR, Gwede CK, Meade CD, Vadaparampil ST, ENRICH/ECHO Working Group (2019). Impact of a web-based reproductive health training program: ENRICH (Educating Nurses about Reproductive Issues in Cancer Healthcare). Psychooncology.

[14] Baptista S, Teles Sampaio E, Heleno B, Azevedo LF, Martins C (2018). Web-based versus usual care and other formats of decision aids to support prostate cancer screening decisions: systematic review and meta-analysis. J Med Internet Res.

[15] Diefenbach MA, Benedict C, Miller SM, Stanton AL, Ropka ME, Wen K, Fleisher LG, Mohamed NE, Hall SJ (2018). Examining the impact of a multimedia intervention on treatment decision-making among newly diagnosed prostate cancer patients: results from a nationwide RCT. Transl Behav Med.

[16] Diefenbach MA, Mohamed NE, Butz BP, Bar-Chama N, Stock R, Cesaretti J, Hassan W, Samadi D, Hall SJ (2012). Acceptability and preliminary feasibility of an internet/CD-ROM-based education and decision program for early-stage prostate cancer patients: randomized pilot study. J Med Internet Res.

[17] Ellison GL, Weinrich SP, Lou M, Xu H, Powell IJ, Baquet CR (2008). A randomized trial comparing web-based decision aids on prostate cancer knowledge for African-American men. J Natl Med Assoc.

[18] Tomko C, Davis KM, Luta G, Krist AH, Woolf SH, Taylor KL (2015). A comparison of web-based versus print-based decision AIDS for prostate cancer screening: participants' evaluation and utilization. J Gen Intern Med.

[19] Cockle-Hearne J, Barnett D, Hicks J, Simpson M, White I, Faithfull S (2018). A web-based intervention to reduce distress after prostate cancer treatment: development and feasibility of the getting down to coping program in two different clinical settings. JMIR Cancer.

[20] Golsteijn RHJ, Bolman C, Peels DA, Volders E, de Vries H, Lechner L (2017). A web-based and print-based computer-tailored physical activity intervention for prostate and colorectal cancer survivors: a comparison of user characteristics and intervention use. J Med Internet Res.

[21] Diefenbach MA, Butz BP (2004). A multimedia interactive education system for prostate cancer patients: development and preliminary evaluation. J Med Internet Res.

[22] Chung C, Cooper SJ, Cant RP, Connell C, McKay A, Kinsman L, Gazula S, Boyle J, Cameron A, Cash P, Evans L, Kim J, Masud R, McInnes D, Norman L, Penz E, Rotter T, Tanti E, Breakspear T (2018). The educational impact of web-based and face-to-face patient deterioration simulation programs: An interventional trial. Nurse Educ Today.

[23] Liaw SY, Chng DYJ, Wong LF, Ho JTY, Mordiffi SZ, Cooper S, Chua WL, Ang ENK (2017). The impact of a Web-based educational program on the recognition and management of deteriorating patients. J Clin Nurs.

[24] Sowan AK, Idhail JA (2014). Evaluation of an interactive web-based nursing course with streaming videos for medication administration skills. Int J Med Inform.

[25] Hallett J, Maycock B, Kypri K, Howat P, McManus A (2009). Development of a Web-based alcohol intervention for university students: processes and challenges. Drug Alcohol Rev.

[26] McRee A, Shoben A, Bauermeister JA, Katz ML, Paskett ED, Reiter PL (2018). Outsmart HPV: Acceptability and short-term effects of a web-based HPV vaccination intervention for young adult gay and bisexual men. Vaccine.

[27] Pugatch J, Grenen E, Surla S, Schwarz M, Cole-Lewis H (2018). Information architecture of web-based interventions to improve health outcomes: systematic review. J Med Internet Res.

[28] Stawarz K, Preist C, Coyle D (2019). Use of smartphone apps, social media, and web-based resources to support mental health and well-being: online survey. JMIR Ment Health.

[29] Speed E, Davison C, Gunnell C (2016). The anonymity paradox in patient engagement: reputation, risk and web-based public feedback. Med Humanit.

[30] Hilton JF, Barkoff L, Chang O, Halperin L, Ratanawongsa N, Sarkar U, Leykin Y, Muñoz RF, Thom DH, Kahn JS (2012). A cross-sectional study of barriers to personal health record use among patients attending a safety-net clinic. PLoS One.

[31] Brown LL, Lustria MLA, Rankins J (2007). A review of web-assisted interventions for diabetes management: maximizing the potential for improving health outcomes. J Diabetes Sci Technol.

[32] Heiniger LE, Smith AB, Olver I, Grimison P, Klein B, Wootten A, Abbott JM, Price MA, McJannett M, Tran B, Stockler MR, Gurney H, Butow PN (2017). e-TC: Development and pilot testing of a web-based intervention to reduce anxiety and depression in survivors of testicular cancer. Eur J Cancer Care (Engl).

[33] Kelders SM, Kok RN, Ossebaard HC, Van Gemert-Pijnen JEWC (2012). Persuasive system design does matter: a systematic review of adherence to web-based interventions. J Med Internet Res.

[34] Miller SM, Shoda Y, Hurley K (1996). Applying cognitive-social theory to health-protective behavior: breast self-examination in cancer screening. Psychol Bull.

[35] Miller SM, Tagai EK, Wen K, Lee M, Hui SA, Kurtz D, Scarpato J, Hernandez E (2017). Predictors of adherence to follow-up recommendations after an abnormal Pap smear among underserved inner-city women. Patient Educ Couns.

[36] Marcus AC, Diefenbach MA, Stanton AL, Miller SM, Fleisher L, Raich PC, Morra ME, Perocchia RS, Tran ZV, Bright MA, CISRC Research Team (2013). Cancer patient and survivor research from the cancer information service research consortium: a preview of three large randomized trials and initial lessons learned. J Health Commun.

[37] Wen K, Miller SM, Stanton AL, Fleisher L, Morra ME, Jorge A, Diefenbach MA, Ropka ME, Marcus AC (2012). The development and preliminary testing of a multimedia patient-provider survivorship communication module for breast cancer survivors. Patient Educ Couns.

[38] Miller SM, Hudson SV, Hui SA, Diefenbach MA, Fleisher L, Raivitch S, Belton T, Roy G, Njoku A, Scarpato J, Viterbo R, Buyyounouski M, Denlinger C, Miyamoto C, Reese A, Baman J (2015). Development and preliminary testing of PROGRESS: a Web-based education program for prostate cancer survivors transitioning from active treatment. J Cancer Surviv.

[39] Tagai EK, Hudson SV, Diefenbach MA, Xu J, Bator A, Marziliano A, Miller SM (2021). Social and medical risk factors associated with supportive needs in the first year following localized prostate cancer treatment. J Cancer Surviv.

[40] Tagai EK, Miller SM, Hudson SV, Diefenbach MA, Handorf E, Bator A, Marziliano A, Kutikov A, Hall SJ, Vira M, Schwartz M, Kim IY, Kim S (2021). Improved cancer coping from a web-based intervention for prostate cancer survivors: A randomized controlled trial. Psychooncology.

[41] Charlson ME, Pompei P, Ales KL, MacKenzie CR (1987). A new method of classifying prognostic comorbidity in longitudinal studies: development and validation. J Chronic Dis.

[42] Miller SM (1987). Monitoring and blunting: validation of a questionnaire to assess styles of information seeking under threat. J Pers Soc Psychol.

[43] Parker RM, Baker DW, Williams MV, Nurss JR (1995). The test of functional health literacy in adults: a new instrument for measuring patients' literacy skills. J Gen Intern Med.

[44] Chew LD, Bradley KA, Boyko EJ (2004). Brief questions to identify patients with inadequate health literacy. Fam Med.

[45] Anderson KO, Dowds BN, Pelletz RE, Edwards WT, Peeters-Asdourian C (1995). Development and initial validation of a scale to measure self-efficacy beliefs in patients with chronic pain. Pain.

[46] Curran SL, Andrykowski MA, Studts JL (1995). Short Form of the Profile of Mood States (POMS-SF): Psychometric information. Psychological Assessment.

[47] Radloff LS (2016). The CES-D Scale. Applied Psychological Measurement.

[48] IBM SPSS software. IBM Corp.

[49] R Core Team (2013). R: A language and environment for statistical computing. R Foundation for Statistical Computing.

[50] Roussi P, Miller SM (2014). Monitoring style of coping with cancer related threats: a review of the literature. J Behav Med.

[51] (2019). Internet/Broadband Fact Sheet. Pew Research Center.

[52] (2003). Internet Health Resources. Pew Research Center.

[53] Bacharach VR, Baumeister AA, Furr RM (2003). Racial and gender science achievement gaps in secondary education. J Genet Psychol.

[54] Voight A, Hanson T, O'Malley M, Adekanye L (2015). The racial school climate gap: within-school disparities in students' experiences of safety, support, and connectedness. Am J Community Psychol.

